# Comparative analysis of chloroplast genomes of cultivars and wild species of sweetpotato (*Ipomoea batatas* [L.] Lam)

**DOI:** 10.1186/s12864-021-07544-y

**Published:** 2021-04-13

**Authors:** Shizhuo Xiao, Pan Xu, Yitong Deng, Xibin Dai, Lukuan Zhao, Bettina Heida, An Zhang, Zhilin Zhou, Qinghe Cao

**Affiliations:** 1 Jiangsu Xuzhou Sweetpotato Research Center/Sweetpotato Research Institute, China Agricultural Academy of Sciences, Xuzhou, 221131 China; 2 College of Pastoral Agriculture Science and Technology, Lanzhou University, Lanzhou, 730020 China; 3 International Potato Center, Av.La Molina 1895, La Molina, Lima, Peru

**Keywords:** Sweetpotato, *Ipomoea*, Chloroplast genome, Comparative analysis, Genetic structure

## Abstract

**Background:**

Sweetpotato (*Ipomoea batatas* [L.] Lam.) is an important food crop. However, the genetic information of the nuclear genome of this species is difficult to determine accurately because of its large genome and complex genetic background. This drawback has limited studies on the origin, evolution, genetic diversity and other relevant studies on sweetpotato.

**Results:**

The chloroplast genomes of 107 sweetpotato cultivars were sequenced, assembled and annotated. The resulting chloroplast genomes were comparatively analysed with the published chloroplast genomes of wild species of sweetpotato. High similarity and certain specificity were found among the chloroplast genomes of *Ipomoea* spp. Phylogenetic analysis could clearly distinguish wild species from cultivars. *Ipomoea trifida* and *Ipomoea tabascana* showed the closest relationship with the cultivars, and different haplotypes of *ycf1* could be used to distinguish the cultivars from their wild relatives. The genetic structure was analyzed using variations in the chloroplast genome. Compared with traditional nuclear markers, the chloroplast markers designed based on the InDels on the chloroplast genome showed significant advantages.

**Conclusions:**

Comparative analysis of chloroplast genomes of 107 cultivars and several wild species of sweetpotato was performed to help analyze the evolution, genetic structure and the development of chloroplast DNA markers of sweetpotato.

**Supplementary Information:**

The online version contains supplementary material available at 10.1186/s12864-021-07544-y.

## Background

Sweetpotato (*Ipomoea batatas* [L.] Lam.) is a globally important food crop, and widely used as an industrial and bioenergy resource [1]. Given its relatively high yields and strong adaptability, this species plays an important role in the food security of developing countries [1, 2]. Sweetpotato belongs to *Ipomoea* genus of Convolvulaceae, which has been the only hexaploid (2n = 6x = 90) species in Convolvulaceae [3]. The genome of this species is highly heterozygous and its genome size has reached 1.5 Gb, leading to a lack of high-quality and complete reference genome sequences [4–7]. To date, the origin and evolution of sweetpotato remains unclear [8]. The modern sweetpotato has been speculated to be the result of an initial cross between a tetraploid progenitor and a diploid progenitor, followed by a second whole-genome duplication [7]. The most probable diploid progenitor of sweetpotato is *Ipomoea trifida*, a view that is supported by the whole genome sequencing data of *I.trifida* [6], however, the tetraploid progenitor is still unknown.

Chloroplasts are key organelles of plants. In addition to their well-known function in photosynthesis, chloroplasts are also involved in important biological processes such as plant immunity and crop quality [9, 10]. The genetic transformation of chloroplasts has become a hotspot in genetic engineering [11]. The chloroplast genome is a closed circular DNA, existing in the form of multiple copies in cells. The chloroplast genome of the higher plants has a highly conserved quadripartite circular structure ranging in size between 115 and 165 kb. Two inverted repeat (IR) sequences divide the entire circular chloroplast genome into a large single copy (LSC) and a small single copy (SSC) [12, 13].

The chloroplast genome contains important genetic information. The coding and non-coding regions of the chloroplast genome have significant differences in the speed of the molecular evolution, and these discrepancies are suitable for the systematic studies of different classes [14]. In addition, the nucleotide substitution rate of the chloroplast DNA (cpDNA) is moderate, and the size of the chloroplast genome is not very large, leading it convenient for sequencing. The chloroplast genomes of various species have good collinearity, allowing easier assembly of amount of chloroplast genomes. These advantages of the chloroplast genome are more conspicuous especially for species with complex nuclear genome, such as sweetpotatoes. Therefore, plastomics approaches based on the chloroplast genome have been developed rapidly in recent years [15–18].

China is the largest sweetpotato producer globally, with an annual yield of 5324.57 tons, accounting for 57.91% of the world’s total yield [19]. In the last century, the sweetpotato varieties widely grown in China were mainly ‘Okinawa 100’ from Japan and ‘Nancy Hall’ from the United States, and their derivatives or progenies, such as Xushu 18, the most widely grown sweetpotato variety in China, which has become the parent of many popular cultivars in China [20, 21]. The genetic background of sweetpotato is relatively narrow in China [20], while the number of accessions has been increasing and thus it is necessary to performed molecular identification and diversity analysis of sweetpotato cultivars. The genetic diversity of sweetpotato has been analysed using molecular markers, such as simple sequence repeats and amplified fragment length polymorphisms [21, 22]. Considering the polyploid nature of sweetpotato the specificity of these markers is not ideal. Sequence-based single nucleotide polymorphism (SNP) and specific length amplified fragment can improve the density of markers [20, 23]. However, the reliability of variant calling is debateable because of the lack of high-quality reference genome. Until the details of nuclear genome can be obtained accurately, using chloroplast genome is a good alternative to analyze the genetic diversity of sweetpotato.

In this study, the chloroplast genomes of 107 sweetpotatoes were sequenced and assembled. Combined with the published chloroplast genomes of eleven wild species, comparative genome, systematic evolution and genetic structure analysis were performed. User-friendly molecular markers were designed based on insertion-deletion (InDel) variants in the chloroplast genome. The results laid the foundation for the study on the plastomics, genetic evolution and precise molecular identification of sweetpotato.

## Results

### Whole genome resequencing and chloroplast genome assembly of 107 sweetpotato cultivars

**Fig. 1:**
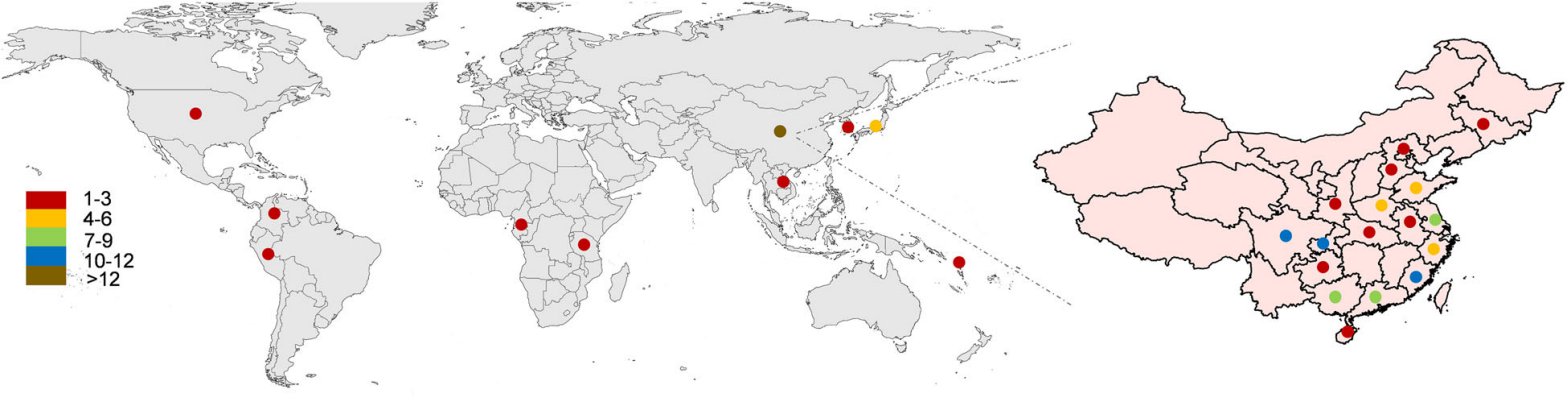
Geographical distribution and number of sweetpotato samples. The different colors represent the number of samples acquired from this region. Global samples on the left and Chinese samples on the right. The maps were drawn using following R packages: “maptools 1.0–2” [24], “maps 3.3.0” [25], “ggmap 3.0.0” [26] and “mapdata 2.3.0” [27]

A total of 107 sweetpotato cultivars were acquired worldwide, of which 92 samples were from various provinces in China (Fig. 1, Table S1). The whole genomes of 107 sweetpotato cultivars were resequenced using NovaSeq 6000 platform, and 2064.03 Gb of raw data were obtained. After filtering was applied, the following data were obtained: 2056.36 Gb of clean data, average of 19.22 Gb for each sample; average sequencing depth, more than 12-fold; Q20, 95.68–97.85%; Q30, 89.22–93.73%; and GC contents, 36.49–39.41%, with an average GC content of 37.54%. (Table S1).

The chloroplast genome of Xushu18 was selected as a reference genome, and the alignment reads were screened for assembly. The average lengths of contigs N50 and N90 were 77,921 bp and 11,343 bp respectively, and the average lengths of scaffolds N50 and N90 reached 85,023 bp and 16,431 bp respectively while the average number of gaps was 1.88 (Table S1). The long scaffolds were selected to be spliced into circular DNA.

### Chloroplast genome structure of sweetpotato

**Fig. 2:**
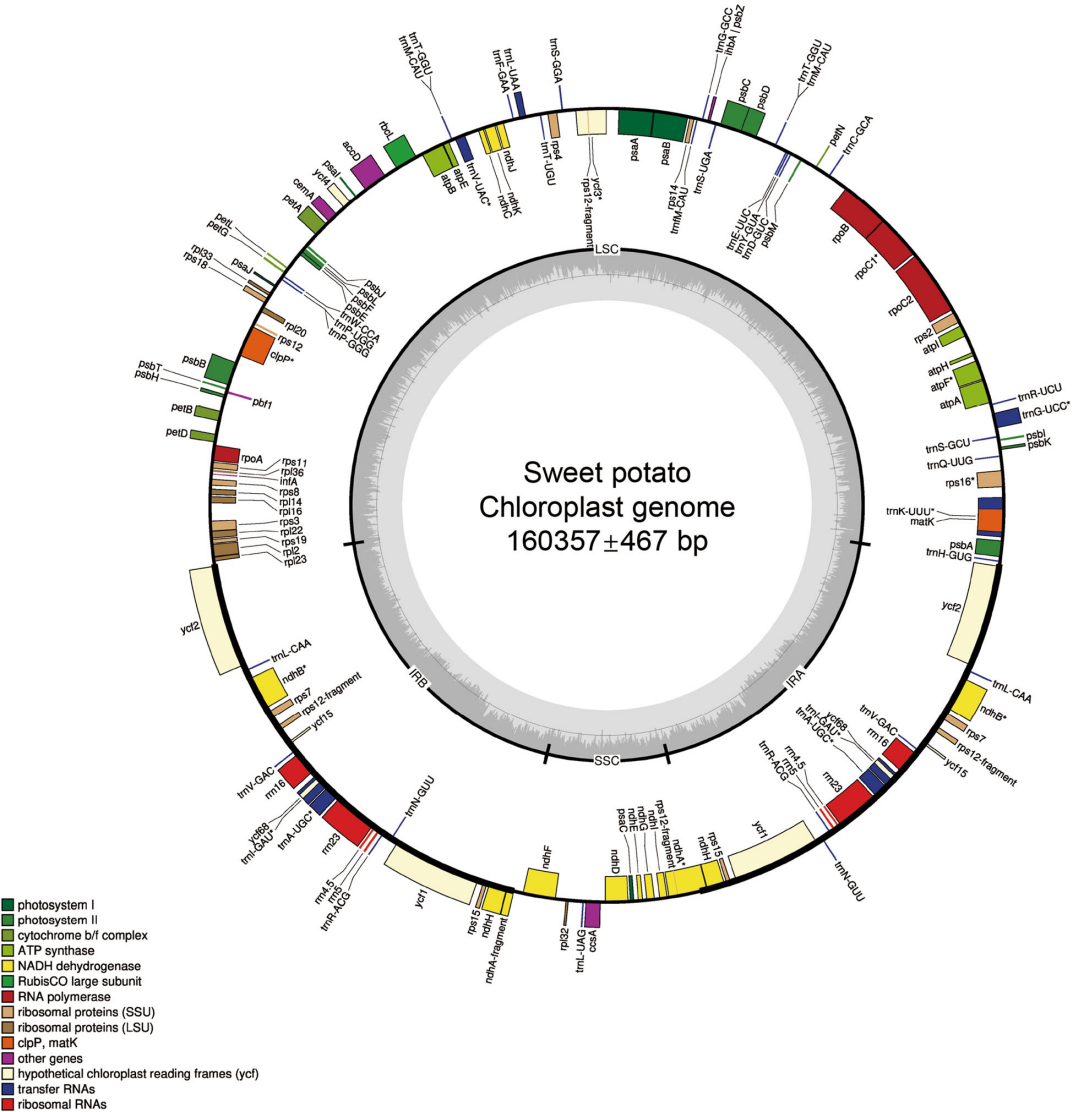
Chloroplast genome map of 107 sweetpotatoes. Genes drawn inside the circle are transcribed clockwise, and those outside the circle are transcribed counterclockwise. The darker gray in the inner circle represents GC content. LSC, large single copy; SSC, short single copy; IR, inverted repeats. The lengths of the chloroplast genomes are marked in the center

The chloroplast genome of sweetpotato has the quadripartite structure typical for most higher plants. The length of these chloroplast genomes varied between 156,888 bp and 161,302 bp, with Med = 87,754 bp and x̅ = 87,791 ± 157 bp (Fig. 2). The LSC (length = 87,589–88,298 bp, Med = 87,754 bp and x̅ = 87,791 ± 157 bp) and SSC (length = 12,047–12,143 bp, Med =12,065 bp and x̅ = 12,068 ± 21 bp) were separated by two IRs (length = 26,923–30,675 bp, Med = 30,226 bp and x̅= 30,220 ± 347 bp). The chloroplast genomes of the sweetpotatoes and the reference cultivar Xushu 18 showed good synteny, which demonstrated the conservation of the chloroplast genome of *I. batatas* (Fig. S1).

A complete chloroplast genome of sweetpotato contained 80 genes encoding protein, of which eight pairs were located in two IR region. A total of 37 tRNAs were found, of which seven were noted in each IR region. In addition, 22 ORFs containing introns were observed, consisting of 14 protein-encoding genes (*ndhA*, *ndhB*, *rps16*, *rpoC1*, *ycf3*, *clpP*, *petB*, *petD*, *rpl16*, *atpF* and two copies of *ndhB* and *rps*) and eight tRNAs (*trnK-UUU*, *trnG-UCC*, *trnL-UAA*, *trnV-UAC* and two copies of *trnI-GAU* and *trnA-UGC*) (Fig. 2).

### Comparative analysis of the *Ipomoea* chloroplast genome

**Fig. 3:**
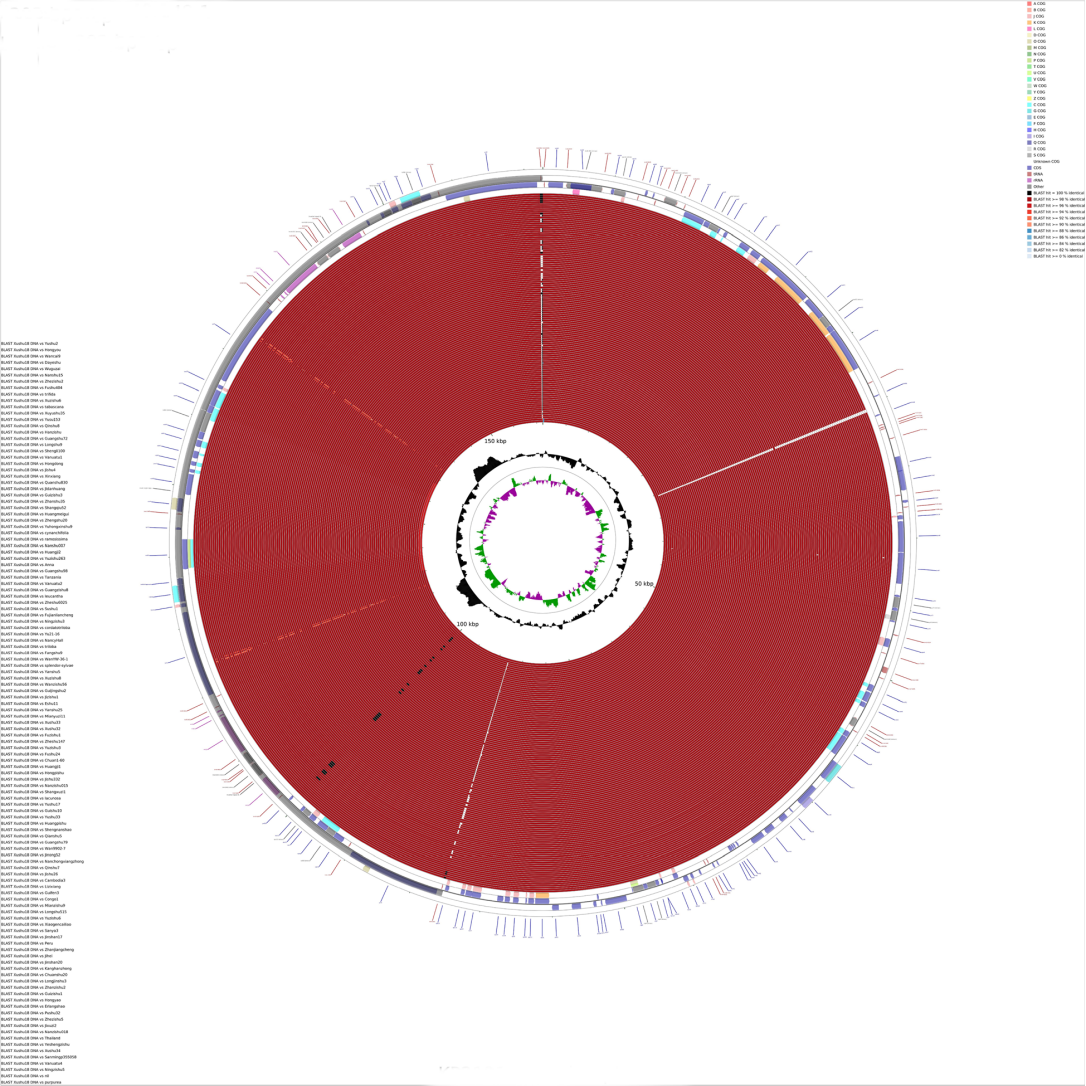
Comparative analysis of chloroplast genomes of 107 sweetpotatoes and 11 wild species. The contents of the feature rings (starting with the outermost ring) are as follows: Ring 1: COG functional categories for forward strand coding sequences; Ring 2: forward strand sequence features; Ring 3: reverse strand sequence features; Ring 4: COG functional categories for reverse strand coding sequences. The next 118 rings show regions of sequence similarity detected by BLAST comparisons conducted by DNA sequence between the reference genome and the 118 chloroplasts genomes. The two innermost rings show GC skew and GC content respectively

The chloroplast genomes of 11 *Ipomoea* species were downloaded from NCBI and used to conduct a comparative chloroplast genome analysis with 107 sweetpotato cultivars (Fig. 3). Among the different cultivars and even different species of the genus *Ipomoea*, the similarity of most nucleotide sequences was higher than 98%. However, some exceptions existed, namely, at approximately 115–136 kb at the LSC, *Ipomoea nil* and *Ipomoea purpurea* showed similarity lower than 98% and higher than 94% compared to other *Ipomoeas* species. This region contained the entire SSC, the *rps15* and *ndhH* ocated in the two IRs. These two wild species belong to the Section *Quamoclit* and are regarded to have relatively distant relationship to the sweetpotato cultivars. The difference in this segment of the chloroplast genomes could also support this hypothesis. Moreover, an obvious low-similarity site existed in two copies of the *ycf1* gene. The *ycf1* gene encoded a protein with unknown function possibly involved in protein transport as a component of a complex [28].

Considering the polymorphism of *ycf1* gene, *ycf1* from some cultivars and 11 wild species was selected, and amino acid (AA) sequences were aligned (Fig. S2). The results demonstrated a repeat region with SEKKSETD sequence as a unit from 1755 AA to 1810 AA on the gene. Four repeats were found in cultivars, five or six repeats in the wild species of Section *batatas*. In addition, seven repeats were found in *I.nil* and *I.purpurea*, and two mutations in *I.purpurea*. Hence, *ycf1* had its specific haplotypes in the cultivars, wild relatives and relatively distant wild species.

### Phylogenetic analysis of *Ipomoea*

**Fig. 4:**
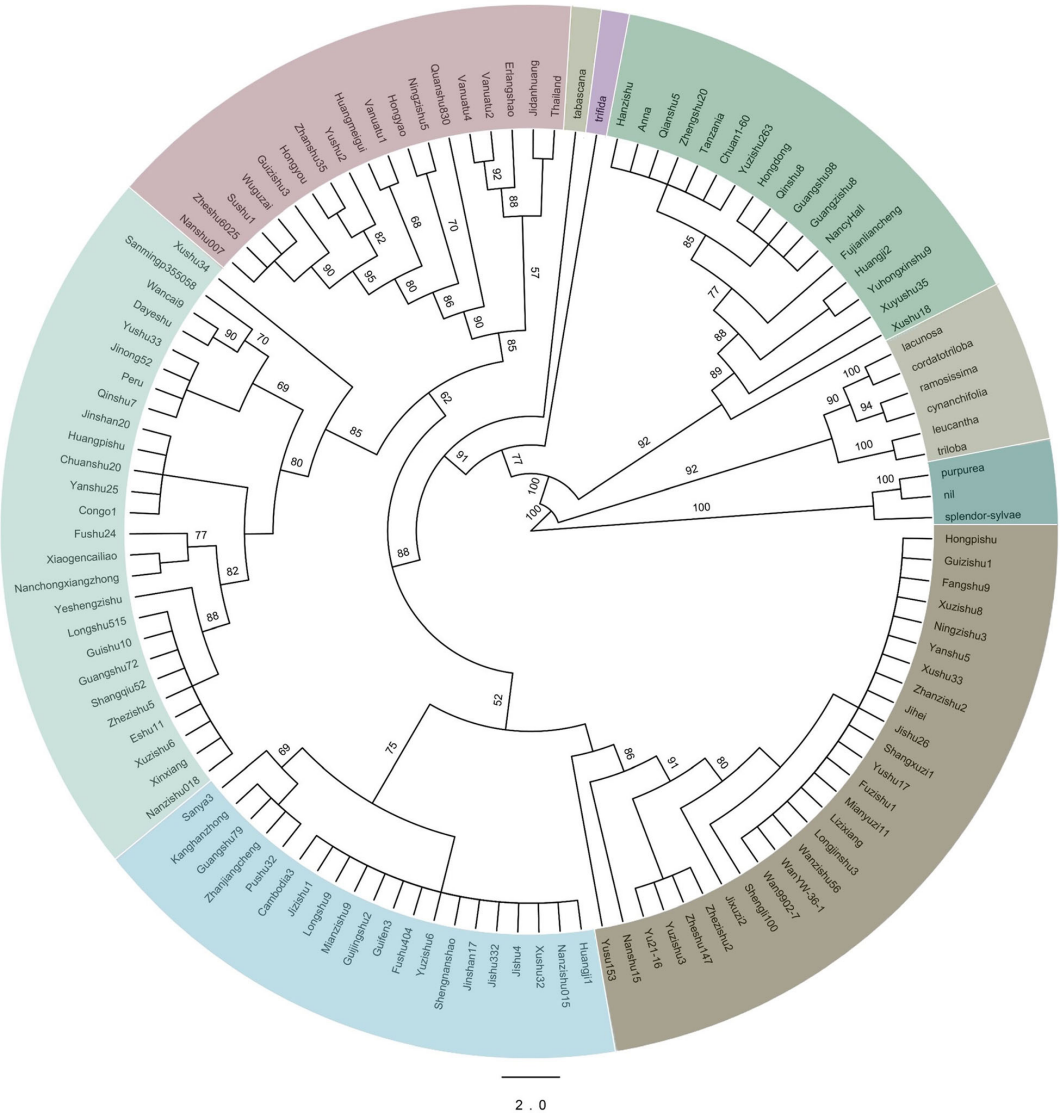
Phylogenetic tree of 107 sweetpotato cultivars and 11 wild species based on single-copy genes of the chloroplast. Different branches are colored differently, branch I, II, IV and V are wild species and the others are cultivars

Phylogenetic analysis of 11 wild species and 107 sweetpotato cultivars was performed based on the single-copy genes annotated in the chloroplast genome with the maximum likelihood (ML) (Fig. 4). These samples were clearly divided into nine branches. Branch I including three wild species (*I.purpurea, I.nil* and *I.splendor-sylvae*) was the farthest from cultivars. Branch II composed six wild species (*I.triloba*, *I.lencantha*, *I.cynanchifolia*, *I.ramosissima*, *I.cordatotriloba* and *I.lacunosa*) all of which belong to section *Batatas*. The section *Batatas* comprises the closest relatives of the sweetpotato cultivars. The other two wild species (*I.trifida* and *I.tabascana*) were classified into two separate branches: branches IV and V, and their relationship with the cultivars were the closest. The diploid species *I.trifida* has long been considered as one of the ancestors of sweetpotato [6]. This phylogenetic analysis may support this perspective.

Xushu18, which used to be the most widely planted cultivar in China, and one of its parents ‘NancyHall’ were classified into branch III. Most cultivars in this branch were relatively related to Xushu18 or its parents. Compared with other branches, branch VI showed greater diversity, and although only 18 cultivars in this branch existed, these cultivars were divided into 9 sub-branches. The cultivars of this branch came from multiple countries, including Japan, Thailand, Vanuatu and some ancient Chinese landrace were also included. Branches VII and IX were the two largest branches containing 26 and 27 sweetpotato cultivars, respectively. Branch VII was dominated by the orange-fleshed sweetpotatoes, accounting for 18 out of 26 cultivars. By contrast, most cultivars of branch IX were purple sweetpotatoes, accounting for 15 out of 27 cultivars. The cultivars of branch VIII mostly originated from the coastal areas of southern China, including three samples from Guangdong Province, three samples from Fujian Province, two samples from Guangxi Province, and two samples from Hainan Province (Fig. 4, Table S1).

A phylogenetic tree based on *ycf1* was also constructed (Fig. S3). The classification of cultivars had no obvious correlation with the phylogenetic tree based on the chloroplast genomes. However, the wild relatives of sweetpotato were still clearly formed a branch, indicating that the gene was representative for the different taxa of *Ipomoea.*

Hence, although the sweetpotato cultivars were divided into different branches, no significant relation of traits such as geography or flesh color and branches could be observed. This result might have been caused by the widespread mutual introduction and cross-fertilization of cultivars between different regions in China.

### Variants calling and genetic structure analysis

**Fig. 5:**
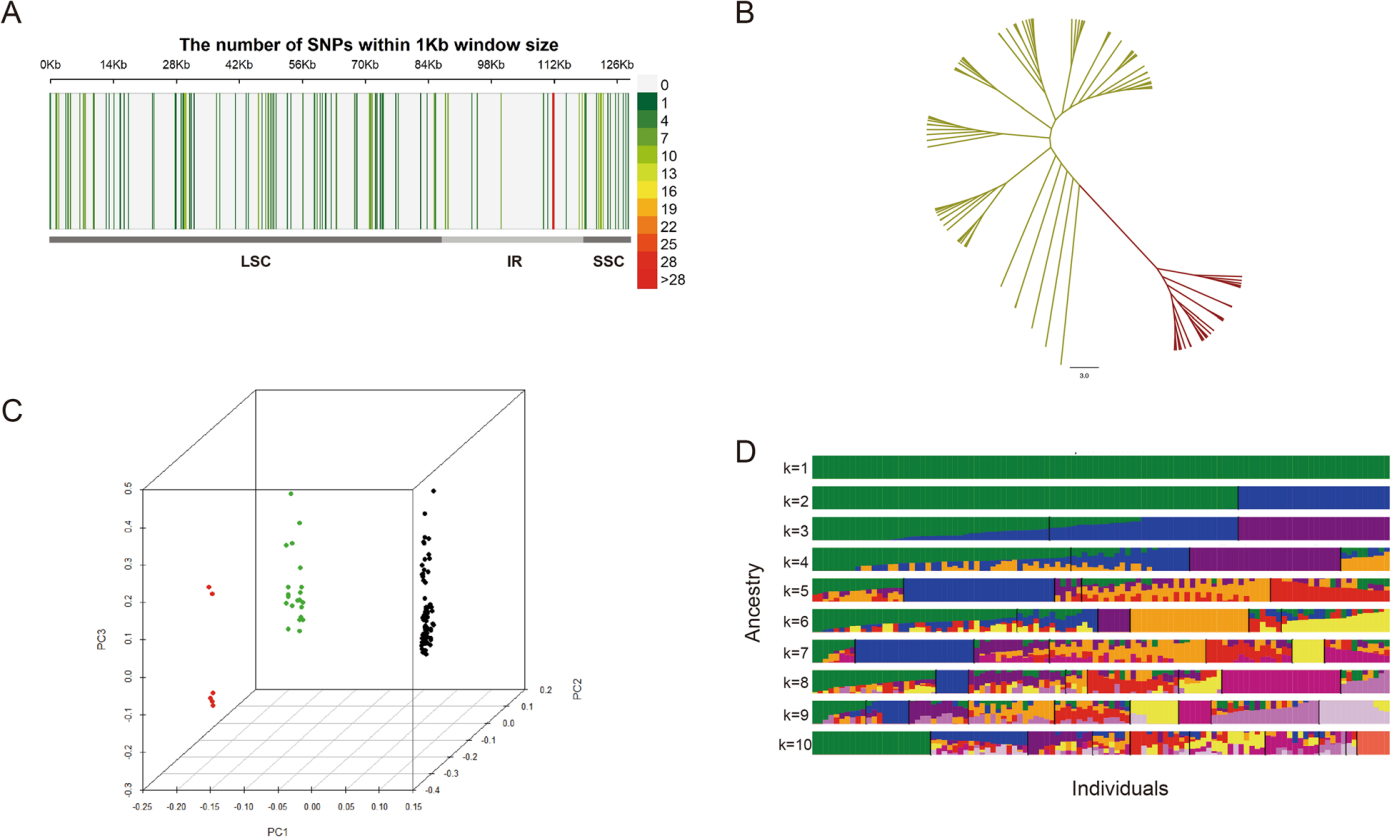
The genetic structure analysis of 107 sweetpotato cultivars based on variation of the chloroplast genome. SNP density distribution on the chloroplast genome (**a**); Phylogenetic tree (**b**); Principal component analysis (**c**); Population structure analysis with K ranging from 1 to 10 (**d**)

Variants on cpDNA of sweetpotato were detected using the chloroplast genome of Xushu18 as reference. A total of 229 mutation sites were screened, including 118 SNPs and 111 InDels (Fig. 5a). Among these variants, 129 variants were located upstream and downstream of the genes (66 SNPs and 63 InDels), three variants in the intergenic regions (all of which were SNPs) and 31 variants were in the ncRNA or introns (25 SNPs and 6 InDels). A total of 66 variants were in the exons (54 SNPs and 12 InDels), of which 25 were non-synonymous variants (Table S2). The gene *ycf1* harboured as many as 31 mutation sites, which corresponded to the results of the comparative genomics analysis (Fig. 3).

The extracted SNPs were used for the genetic structure analysis. The construction of the phylogenetic tree, principal component analysis (PCA) and population structure analysis were performed (Fig. 5). The phylogenetic tree showed that two major groups were clearly clustered (Fig. 5b). When three principal components were used, the 107 cultivars could be divided into three groups, and the most of the samples were divided into the two largest groups, while the smallest group just consisted of 6 samples (Fig. 5c). The population structures were analyzed with the K value ranging from 1 to 10, and the population were clearly separated with K = 2 (Fig. 5d). The cross validation (CV) error was also the lowest with K = 2 (Fig. S4). Taken together, the findings suggested that it was better to divide the sweetpotato population into two groups.

### Development of chloroplast DNA markers

Based on the InDels detected on the chloroplast genome, site variations with base number not lower than three were selected to design the cpDNA markers. A total of 20 pairs of amplification primers were designed. The lengths of these primer ranged from 20 bp to 26 bp, and the Tm scores were between 57.47 °C and 60.42 °C. The maximum Tm difference between the forward and reverse primers was 1.95 °C. The lengths of products were mostly between 130 bp and 195 bp, except for Ibcp-15 with its product reaching 300 bp (Table S3). Of the cpDNA markers, 13 were from the LSC, four were from the IR regions and three were from the SSC.

**Fig. 6:**
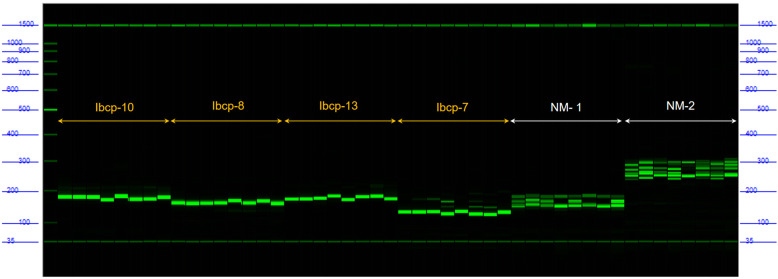
Capillary electrophoresis using cpDNA markers. The four groups on the left were products amplified by cpDNA markers (Ibcp-10, Ibcp-8, Ibcp-13, Ibcp-7), and the other two groups on the right were products amplified by nuclear markers (NM-1, NM-2)

To verify the availability of the cpDNA markers, eight sweetpotato cultivars were selected randomly, and their DNAs were extracted as templates. Capillary electrophoresis was carried out after PCR. Compared with the nuclear DNA markers, the bands of cpDNA marker were simplex, distinct and more readable (Fig. 6). Moreover, these markers showed good polymorphism among the samples. Therefore, these excellent cpDNA markers could provide a powerful tool for the analysis of sweetpotato genetic diversity or the construction of fingerprints of sweetpotato cultivars.

## Discussion

In this study, 107 sweetpotato cultivars were resequenced. Given the genome size of sweetpotato, the resequencing of abundant samples is a labourious task. At present, available reference genomes of the genus *Ipomoea* are from the diploid wild relatives: *I.trifida* [4, 6, 29], *I.triloba* [6], and *I.nil* [5], and from the haplotype genome of cultivar Taizhong 6 [7]. The application of resequenced natural populations is limited by the lack of high-quality reference genomes. The de novo sequencing of the sweetpotato cultivar Xushu18 promoted as a collaborative effort by China, Japan and South Korea will be completed in the near future [30, 31]. It is an opportunity for the genome-wide association analysis or pan-genome analysis using the resequenced population of sweetpotatoes.

The cpDNA participates in some important physiological processes in plants. The information carried by the cpDNA can help in the more comprehensive understanding of the plant genetics and evolution. In this study, cpDNA reads were extracted from the massive DNA sequences, homology assembly and annotation were accomplished and 107 high-quality chloroplast genomes were obtained. Given the complexity of the sweetpotato nuclear genome and the difficulty to obtain nuclear genome information, a comprehensive understanding of the chloroplast genome is a valuable supplement.

The assembled chloroplast genomes of sweetpotatoes were all of the typical quadripartite circular structure, which was highly conserved among species in the the genus *Ipomoea*, especially for the nucleotide sequence with a similarity of more than 98%. Nevertheless, *I.nil* and *I.purpurea* could be distinguished distinctly by the region of 115–136 kb in the LSC (Fig. 3). This characteristic may be the reason why *I.nil* and *I.purpurea* were divided into a single branch in the phylogenetic analysis (Fig. 4).

Whether comparative genomics analysis or variants calling, it has been proved that *ycf1* harboured abundant mutations (Figs. 3 and 5a, Table S2). Previous studies have showed that *ycf1* had a relatively fast evolutionary rate among different species. Thus, this gene was used as a cpDNA barcode to identify different species [32, 33]. In this study, *ycf1* not only greatly varied among different species but also among different varieties of sweetpotato (Fig. 3). Given the considerable variation in *ycf1*, *ycf1* has become a pseudogene in some species, and the loss of *ycf1* is common in plants such that whether *ycf1* was indispensable is controversial [34–36]. The results in this study showed that *ycf1* in sweetpotato had a complete opening reading frame and could normally encode proteins. In addition, its AA sequence could be used to distinguish sweetpotatoes from its wild relatives (Figs. S2 and S3).

Based on the single-copy genes of the chloroplast genome, eleven wild species and 107 cultivars of sweetpotato were divided into nine branches (Fig. 4). The wild species and cultivars were clearly separated, with four branches composed of wild species and five branches composed of cultivars. Among the wild species, *I.tabascana* and *I.trifida* were divided into two separate branches, and these two wild species were more closely related to the cultivars than any other wild species. The origin of sweetpotato cultivars has long been ambiguous, but there is broad consensus that *I.trifida* is one of the ancestors of sweetpotato, which was confirmed by cytological markers and chromosomal markers [8, 37]. It was also confirmed that *I.trifida* was a wild species with relatively close relationship with cultivars using chloroplast comparative genome in this study (Fig. 4). Yet whether a tetraploid participated during evolution and which tetraploid participated remain controversial. Although this study could not fully explain the issue, the result that the tetraploid species of *I.tabascana* formed a branch close to the cultivars may provide some useful information for the evolution of sweetpotato.

Genetic diversity and population structure of sweetpotato cultivars have been studied using chromosomal molecular markers before. 38 SSR and 62,363 SNPs were used to analyze the genetic diversity and population structure of Chinese sweetpotato accessions respectively, and these sweetpotato varieties were divided into 3 groups [20, 38], which was consistent with the results of PCA in this study (Fig. 5c). Yet there were differences between trees constructed by single-copy genes and variants on chloroplast genome (Figs. 4 and 5b), which may be caused by the differentiation of the evolutionary rate of coding region and the non-coding region on chloroplast genome. That there were only 25 non-synonymous variations among the 229 variation sites also supported this view (Table S2). In addition, some cultivars were not well distinguished at the chloroplast genome level (Fig. 4), which indicated that the chloroplast genome of sweetpotato was intraspecific conserved.

DNA molecular markers are the basis of genetic diversity assessment and molecular fingerprint construction. The sweetpotato is a hexaploidy hence multiple copies and heterozygous loci lead to the very poor usability of common nuclear markers. The amplified positions of the genome were not clear and specific, such that a pair of primers could even produce as many as 20 bands during electrophoresis, leading to poorly readable electrophoresis results. In this study, 20 cpDNA markers were designed according to InDel calling. The specificity of the primers was ensured by preventing their binding to anywhere in the nuclear DNA. Capillary electrophoresis was conducted. Compared with the nuclear markers, the products of the cpDNA markers of sweetpotato showed simplex, distinct, good specificity and high readability characteristics (Fig. 6). cpDNA markers are powerful tools for analysing the sweetpotato genetic diversity or constructing the fingerprints of sweetpotato cultivars. In addition to InDels, abundant SNPs were also found in the cpDNA (Table S2), which can be designed as dCAP markers complementing cpDNA markers of sweetpotato. These results will help improve the homogeneity of the sweetpotato cultivars in China.

## Conclusion

In the present study, the chloroplast genomes of 107 sweetpotato cultivars were sequenced, assembled and annotated. Comparative analysis of the chloroplast genome of 107 cultivars and wild species of sweetpotato was performed. The sweetpotato cultivars and their wild species maintained a high similarity in the chloroplast genome. The cultivars and wild species could be clearly distinguished by the chloroplast genome. *I.trifida* and *I.tabascana* had the closest relationship with the cultivars and may have been involved in the evolution of sweetpotato. The sweetpotato cultivars were obviously grouped into several populations, but without significant relationship with the geographic origin or flesh color. The cpDNA markers designed based on the variation in the chloroplast genome showed significant advantages compared with traditional nuclear markers. The designed marker could be useful for the genetic diversity analysis and molecular identification of sweetpotato cultivars.

## Methods

### Plant materials and resequencing

A total of 107 cultivars were acquired worldwide and conserved in the National Sweetpotato Genebank in Xuzhou, China. All sweetpotato germplasm resources were public varieties or landraces. Among these germplasm resources, 92 were from China, and the others were from the United States, Cambodia, Congo, Japan, Peru, South Korea, Tanzania, Thailand and Vanuatu (Table S1). Fresh leaves of these cultivars were sampled and ground into powder using liquid nitrogen. The total DNA was extracted using the CTAB method [39]. DNA purity was checked using the NanoPhotometer® spectrophotometer (IMPLEN, CA, USA). DNA concentration was measured using Qubit® DNA Assay Kit in Qubit® 2.0 Fluorometer (Life Technologies, CA, USA). A total of 700 ng DNA per sample was used as input material for the DNA sample preparations. Sequencing libraries were generated using NEB Next® Ultra DNA Library Prep Kit for Illumina® (NEB, USA) following the manufacturer’s recommendations and index codes were added to attribute sequences to each sample. DNA was purified using AMPureXP system (BeckmanCoulter, Beverly, USA). After the adenylation of 3′ ends of DNA fragments, the NEB Next Adaptor with hairpin loop structure were ligated to prepare for hybridization. Then electrophoresis was used to select the DNA fragments with specific length. 3 μL USER Enzyme (NEB, USA) was used with size-selected, adaptor-ligated DNA at 37 °C for 15 min followed by 5 min at 95 °C before PCR. Then PCR was performed with Phusion High-Fidelity DNA polymerase, Universal PCR primers and Index (X) Primer. Finally, the PCR products were purified (AMPure XP system) and library quality was assessed on the Agilent Bioanalyzer 2100 system. The qualified library was used to sequence on the NovaSeq 6000 platform. The insert should be 350 bp and 150 bp paired-end sequencing was generated.

### Assembly and annotation of chloroplast genomes of sweetpotatoes

Quality control of the sequencing data was conducted using fastp [40]. HISAT2 [41] was used to align the reads screened to the reference chloroplast genome [42]. The aligned reads were used to splice into scaffolds by SPAdes [43]. Synteny analysis between scaffolds and reference chloroplast genome was performed by MUMmer4.0 [44]. Then high-quality scaffolds were selected to assemble circular DNA molecules. Homology annotations were conducted online [45]. The module GeSeq [46] was used to annotate the circular DNA and the results were manually optimized. Another module OGDRAW [47] was used to draw the map.

### Comparative genomic analysis of *Ipomoea*

The chloroplast genome sequences of 11 wild species of *Ipomoea* (*Ipomoea trifida*, *Ipomoea tabascana*, *Ipomoea triloba*, *Ipomoea cordatotriloba*, *Ipomoea cynanchifolia*, *Ipomoea splendor-Sylvae*, *Ipomoea ramosissima*, *Ipomoea leucantha*, *Ipomoea lacunosa*, *Ipomoea nil*, *Ipomoea purpurea*) [48] and cultivar Xushu18 [49] were downloaded from the NCBI [50]. The GeneBank files of the 11 wild species and 107 cultivars were imported to the software CGView Comparison Tool [51], and the script “build_blast_atlas.sh” was used to automatically create maps for nucleotide (blastn) comparison. AA sequences of *ycf1* were aligned by Geneious Basic 4.8.5 [52].

### Construction of phylogenetic tree

To identify gene families, the OrthoFinder (v 2.3.14) pipeline [53] was sequentially applied to the ten genomes with all-to-all BLASTP (E-value ≤1e− 5), reciprocity best hit, pairs connected by orthology and in-paraolgy, normalize the E-value and cluster pairs by OrthoFinder. Finally, genes were classified into orthologues, paralogues and single copy orthologues (only one gene in each species). To construct the phylogenetic tree, single-copy orthologous genes were used; each gene family nucleotide sequence was aligned using Mafft, and the phylogenetic tree was built with both the maximum likelihood and the Bayesian inference (BI) using FastTree [54] and MrBayes [55]. The cladograms of the two methods were compared, and we considered that the evolutionary tree constructed by the ML method was more fit (Fig. 4 and Fig. S5). The phylogenetic tree was visualized and modified by Figtree [56].

### Variants calling

Bowtie2 [57] was used to align the reads of clean data to the reference chloroplast genome. Variant calling was performed using SAMtools and BCFtools [58, 59]. Then the SNPs and InDels were filtered using VCFtools [60] with a missing rate lower than 50%, a minor allele count higher than 3 and a minor allele frequency higher than 0.05. The effect of variants was evaluated by ANNOVAR [61].

### Phylogenetic tree, population structure analysis and PCA based on variants

Filtered SNPs were used to analyse the population structure. CV errors were assessed using ADMIXTURE [62] with default parameters from K = 1 to K = 10. Visualization was conducted by R package (barplot). PCA was conducted by Plink [63], and 3D graph was drawn by R package (scatterplot3d). FastTree was selected for the construction of phylogenetic tree by ML method. Figtree was used for visualization.

### Development and verification of cpDNA markers

The InDels with of base numbers differing by more than three were selected to design the amplification primers. Multiple pairs of primers were simultaneously designed by Primer3 [64, 65]. The parameters were set as follows: lengths of products, less than 200 bp; Tm scores, from 58 to 64 °C; differences of Tm scores between forward and reverse primers, less than 2 °C; GC content, from 35 to 65%; and lengths of primers distributed between 20 bp and 26 bp. The primers with highest score were selected for the nucleotide sequence synthesis by the Sangon Biotech corporation. Eight sweetpotato cultivars were randomly selected to extract the DNA as templates for the general PCR using the designed primers, and two nuclear markers were set as controls. Capillary electrophoresis was performed on the Fragment Analyzer system (AATI, USA) after PCR, and the bands were read on a computer.

## Supplementary Information

**Additional file 1: Fig. S1.** Synteny analysis of the chloroplast genome between cultivar samples and Xushu18

**Additional file 2: Fig. S2.** Alignment of parts of the ycf1 amino acid sequences among some cultivars and wild species of sweetpotato. The first five rows were cultivars, the next nine rows were wild species of section *Batatas* and the last two rows were wild species of section *Quamoclit.*

**Additional file 3: Fig. S3.** Phylogenetic tree of 107 sweetpotatoes and 11 wild species based on *ycf1.* Branch dyed red was wild species of section *Batatas.*

**Additional file 4: Fig. S4.** The CV error with different K value.

**Additional file 5: Fig. S5.** The phylogenetic tree based on single-copy genes by BI method.

**Additional file 6: Table S1.** The details of 107 sweetpotato cultivars.

**Additional file 7: Table S2.** The variations on cpDNA.

**Additional file 8: Table S3.** The cpDNA markers designed based on Indels on cpDNA

## Data Availability

The sequencing data of 107 cultivars using for analysis is deposited in the NCBI, and the accession number is PRJNA715261. The chloroplast genome data of 11 wild species of *Ipomoea* and cultivar Xushu18 used for analysis could be obtained from NCBI, and their accession numbers are as follow: *I.trifida*, MH173261; *I.triloba*, MH173262; *I.leucantha*, NC_041208; *I.tabascana*, NC_041207; *I.splendor-sylvae*, NC_041206; *I.ramosissima*, NC_041205; *I.cordatotriloba*, NC_041204; *I.cynanchifolia*, NC_041203; *I.lacunosa*, MH173257; *I.nil*, AP017304; *I.purpurea*, EU118126; Xushu 18, NC_026703.

## Abbreviations

SNP: Single nucleotide polymorphism
PCR: Polymerase chain reaction
IR: Inverted repeat
LSC: Large single copy
SSC: Small single copy
InDel: Insert and delete
PCA: Principal component analysis
cpDNA: Chloroplast DNA
CDS: Coding sequence
ORF: Opening reading frame
