# Correction to: Comparative analysis of chloroplast genomes of cultivars and wild species of sweetpotato (*Ipomoea batatas* [L.] Lam)

**DOI:** 10.1186/s12864-021-07684-1

**Published:** 2021-05-20

**Authors:** Shizhuo Xiao, Pan Xu, Yitong Deng, Xibin Dai, Lukuan Zhao, Bettina Heider, An Zhang, Zhilin Zhou, Qinghe Cao

**Affiliations:** 1 Jiangsu Xuzhou Sweetpotato Research Center/Sweetpotato Research Institute, China Agricultural Academy of Sciences, Xuzhou, 221131 China; 2 College of Pastoral Agriculture Science and Technology, Lanzhou University, Lanzhou, 730020 China; 3 International Potato Center, Av.La Molina 1895, La Molina, Lima, Peru

**Correction to: BMC Genomics 22, 262 (2021)**

**https://doi.org/10.1186/s12864-021-07544-y**

Following publication of the original article [1], the author’s reported an error in the authorship list.

The name of Bettina Heider was incorrectly given as ‘Bettina Heida’. The original article has been corrected.
