# Supplementary figures and images for: Comparative analysis of chloroplast genomes of cultivars and wild species of sweetpotato (Ipomoea batatas [L.] Lam)
Source: BMC Genomics. 2021 Apr 13;22:262. doi: 10.1186/s12864-021-07544-y (PMC8042981; doi:10.1186/s12864-021-07544-y)

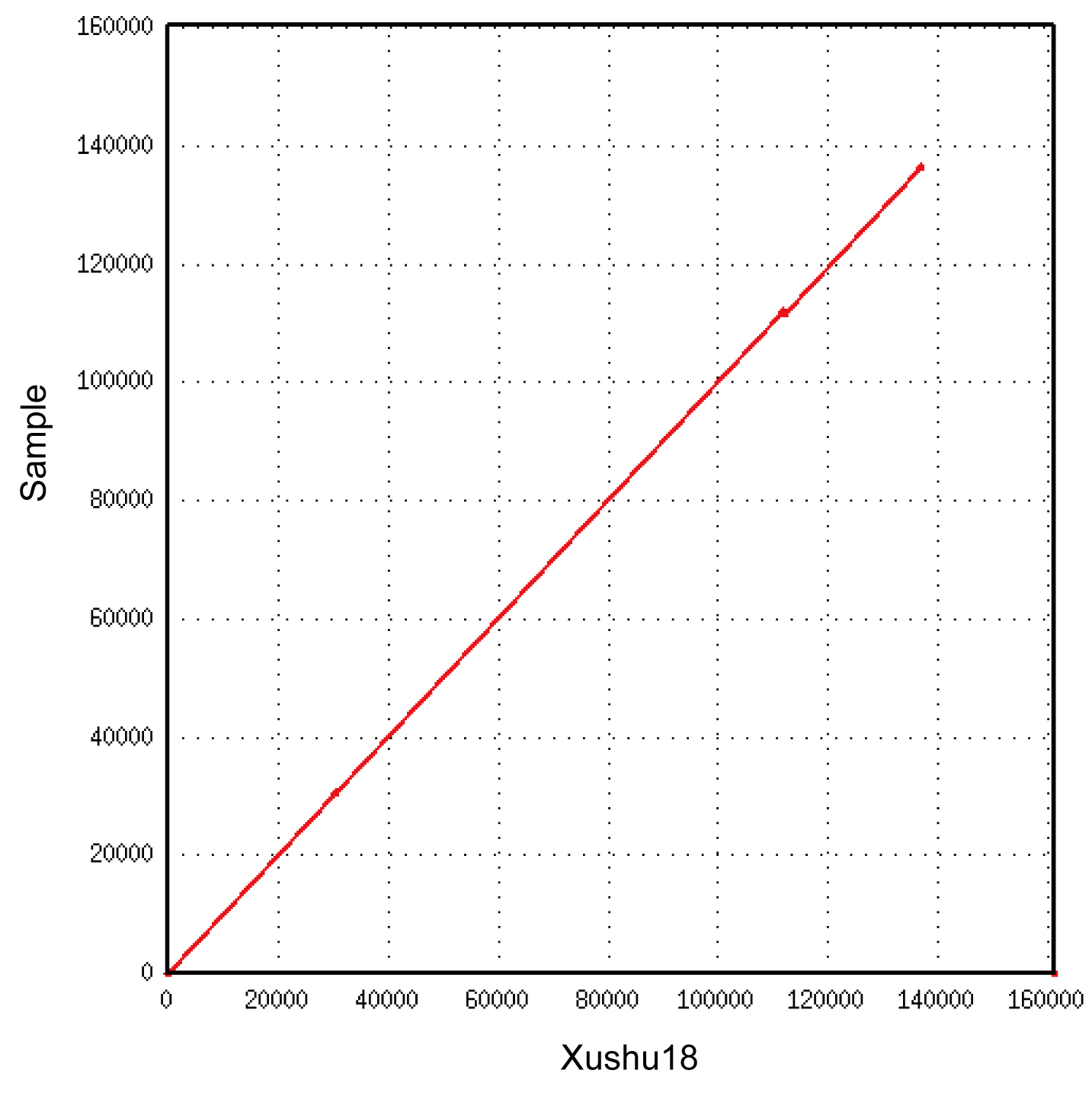

Supplement: Supplementary file 1 — Additional file 1: Fig. S1. Synteny analysis of the chloroplast genome between cultivar samples and Xushu18 [file 12864_2021_7544_MOESM1_ESM.jpg]

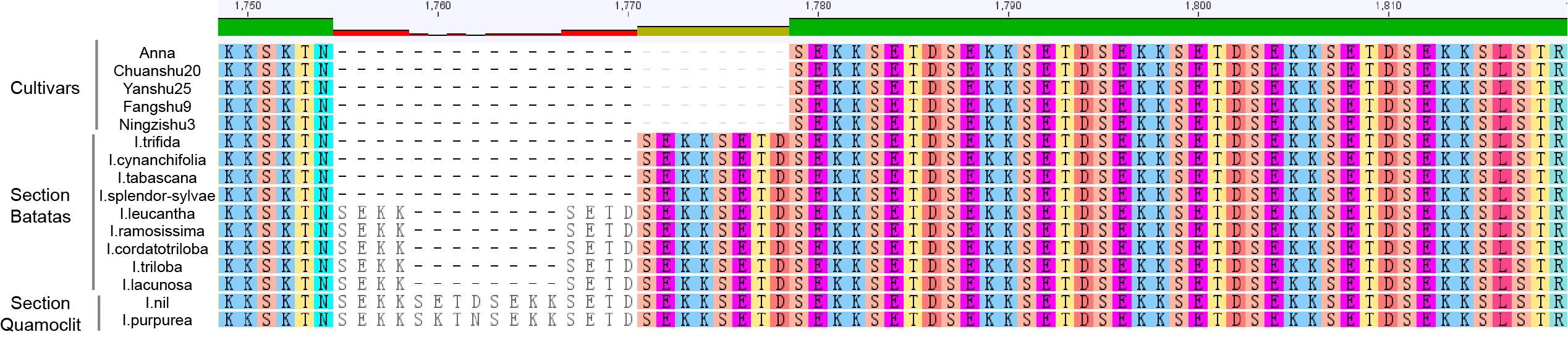

Supplement: Supplementary file 2 — Additional file 2: Fig. S2. Alignment of parts of the ycf1 amino acid sequences among some cultivars and wild species of sweetpotato. The first five rows were cultivars, the next nine rows were wild species of section Batatas and the last two rows were wild species of section Quamoclit. [file 12864_2021_7544_MOESM2_ESM.jpg]

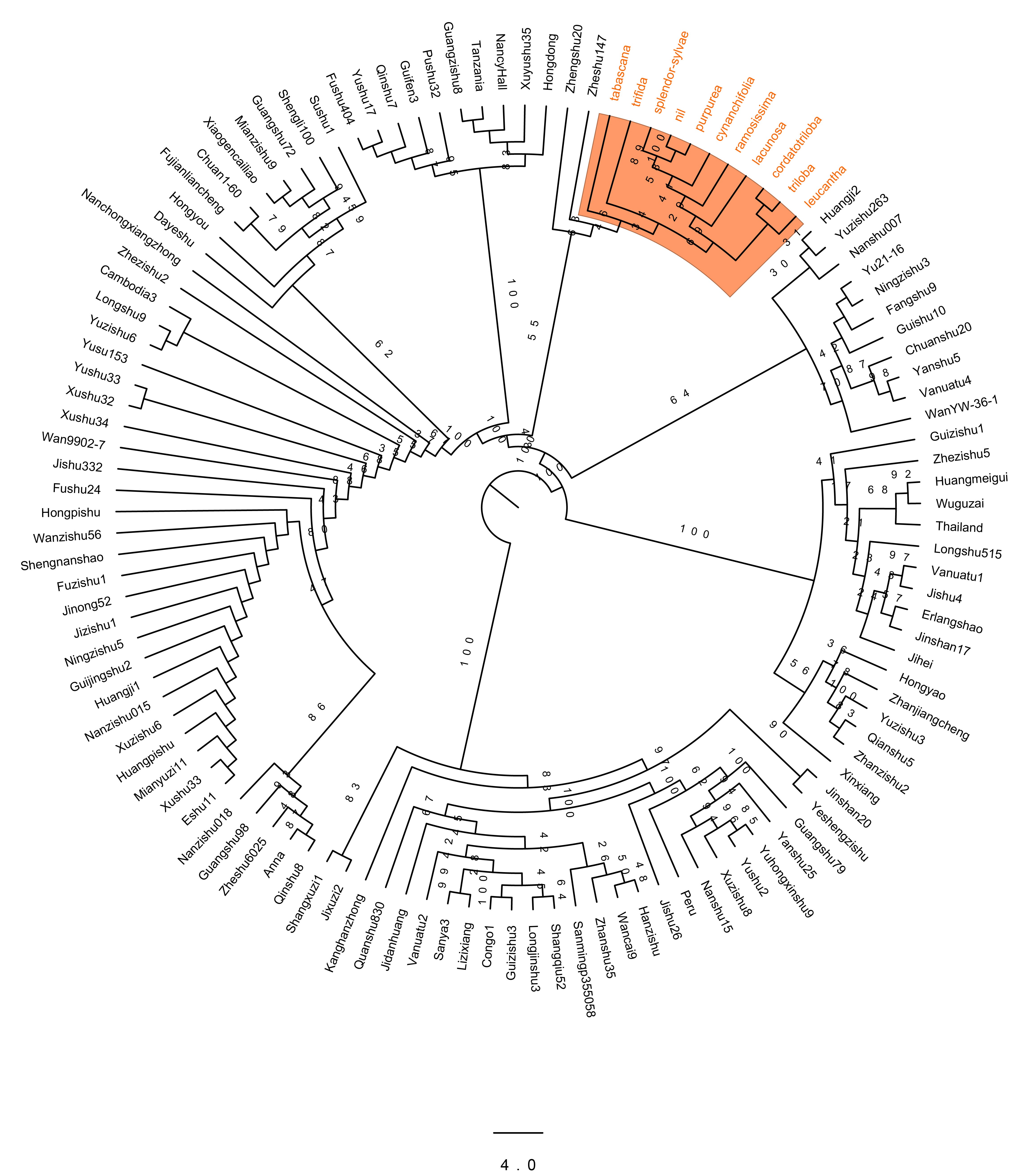

Supplement: Supplementary file 3 — Additional file 3: Fig. S3. Phylogenetic tree of 107 sweetpotatoes and 11 wild species based on ycf1. Branch dyed red was wild species of section Batatas. [file 12864_2021_7544_MOESM3_ESM.jpg]

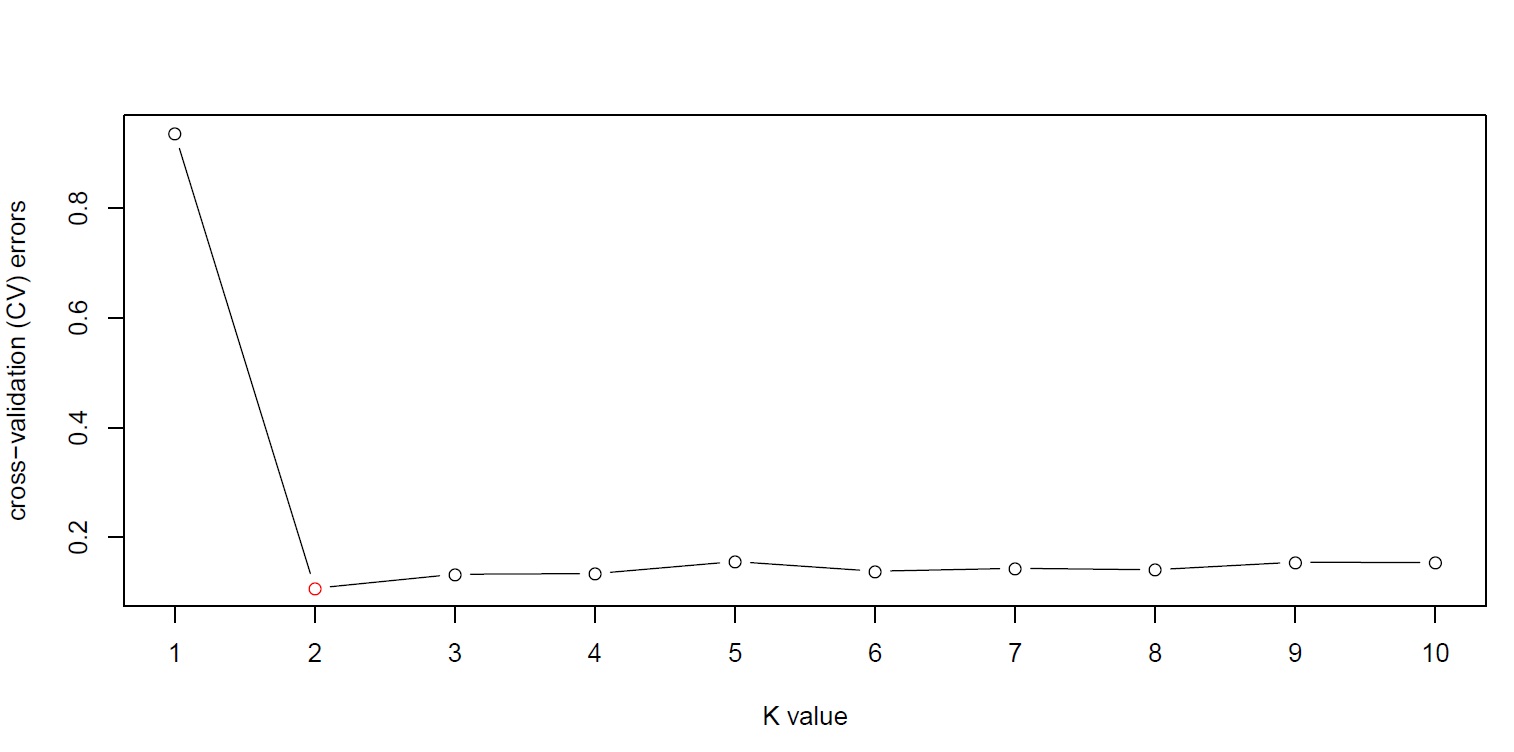

Supplement: Supplementary file 4 — Additional file 4: Fig. S4. The CV error with different K value. [file 12864_2021_7544_MOESM4_ESM.jpg]

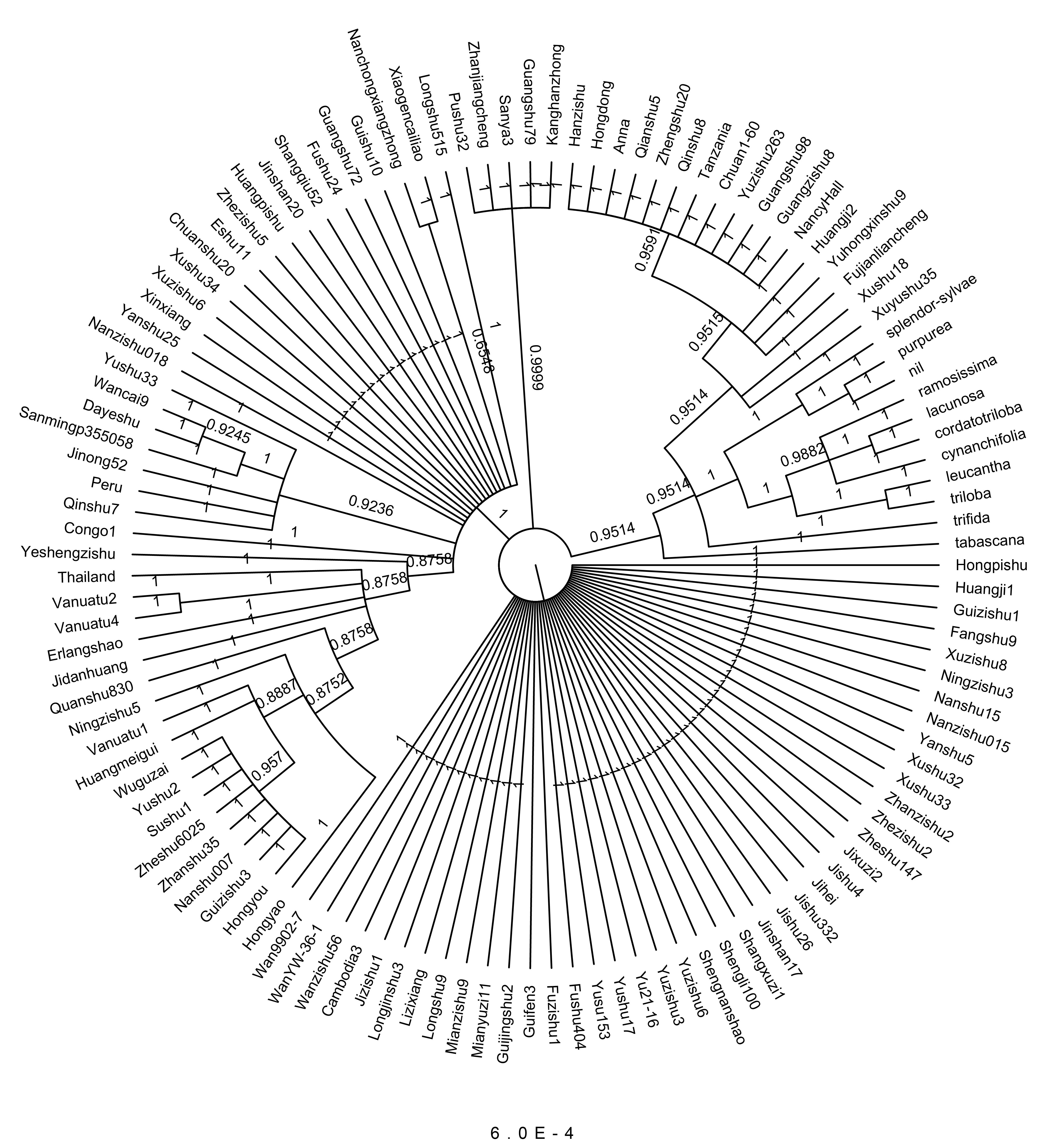

Supplement: Supplementary file 5 — Additional file 5: Fig. S5. The phylogenetic tree based on single-copy genes by BI method. [file 12864_2021_7544_MOESM5_ESM.jpg]
